# The respiratory chain inhibitor rotenone affects peroxisomal dynamics via its microtubule-destabilising activity

**DOI:** 10.1007/s00418-017-1577-1

**Published:** 2017-05-18

**Authors:** Josiah B. Passmore, Sonia Pinho, Maria Gomez-Lazaro, Michael Schrader

**Affiliations:** 1 College of Life and Environmental Sciences, Biosciences, University of Exeter, Geoffrey Pope Building, Stocker Road, Exeter, EX4 4QD UK; 2 Centre for Cell Biology & Department of Biology, University of Aveiro, Aveiro, Portugal; 3 Instituto de Investigação e Inovação em Saúde (i3S), Instituto de Engenharia Biomédica (INEB), University of Porto, Porto, Portugal

**Keywords:** Peroxisomes, Mitochondria, Rotenone, ROS, Organelle cooperation

## Abstract

Peroxisomes and mitochondria in mammalian cells are closely linked subcellular organelles, which maintain a redox-sensitive relationship. Their interplay and role in ROS signalling are supposed to impact on age-related and degenerative disorders. Whereas the generation of peroxisome-derived oxidative stress can affect mitochondrial morphology and function, little is known about the impact of mitochondria-derived oxidative stress on peroxisomes. Here, we investigated the effect of the mitochondrial complex I inhibitor rotenone on peroxisomal and mitochondrial membrane dynamics. We show that rotenone treatment of COS-7 cells alters peroxisome morphology and distribution. However, this effect is related to its microtubule-destabilising activity rather than to the generation of oxidative stress. Rotenone also induced alterations in mitochondrial morphology, which—in contrast to its effect on peroxisomes—were dependent on the generation of ROS but independent of its microtubule-active properties. The importance of our findings for the peroxisome-mitochondria redox relationship and the interpretation of in cellulo and in vivo studies with rotenone, which is widely used to study Parkinson’s disease, are discussed.

## Introduction

Peroxisomes are ubiquitous and dynamic single membrane-bound organelles in eukaryotic cells, that—similar to mitochondria—show an oxidative type of metabolism. Peroxisomes perform important functions in hydrogen peroxide and lipid metabolism rendering them essential for human health and development (Wanders and Waterham 2006). To fulfil their multiple functions, peroxisomes need to communicate and cooperate with other organelles, including mitochondria, the endoplasmic reticulum (ER) or lipid droplets (Schrader et al. 2013, 2015a, b; Gao and Goodman 2015; Costello et al. 2017). Over the years, substantial evidence has been provided for a close functional interplay between peroxisomes and mitochondria (the so-called “peroxisome-mitochondria connection”) which impacts on human health and development (Schrader and Yoon 2007; Camões et al. 2009; Schrader et al. 2015a). Peroxisomes and mitochondria in mammalian cells cooperate metabolically in the breakdown of fatty acids by β-oxidation (reviewed in Wanders et al. 2016); they maintain a redox-sensitive relationship (reviewed in Lismont et al. 2015), share key components of the membrane fission machineries (reviewed in Schrader et al. 2012, 2016), cooperate in anti-viral signalling (Dixit et al. 2010; Odendall et al. 2014) and may depend on each other for biogenesis and functionality (Peeters et al. 2011, 2015; Mohanty and McBride 2013; Sugiura et al. 2017). Of particular interest are their redox-sensitive relationship and their interplay and communication with respect to ROS signalling, which may impact on age-related and degenerative disorders (Koepke et al. 2008; Ivashchenko et al. 2011; Walton and Pizzitelli 2012; Titorenko and Terlecky 2011; Beach et al. 2012; Fransen et al. 2013; Nordgren and Fransen 2014). The generation of oxidative stress in peroxisomes has been shown to impact on mitochondrial morphology and function (Ivashchenko et al. 2011; Wang et al. 2013). However, little is known about the impact of mitochondria-derived oxidative stress on peroxisomes. Here, we have examined the effect of the potent complex I inhibitor rotenone on peroxisomal and mitochondrial membrane dynamics. We show that although rotenone has an impact on peroxisome morphology and distribution, this effect is related to its microtubule-destabilising activity rather than to oxidative stress. On the other hand, rotenone induced alterations in mitochondrial morphology, which were dependent on the generation of ROS but independent of its microtubule-active properties.

## Materials and methods

### Antibodies

Antibodies were used as follows: rabbit polyclonal antibody against PEX14 (1:1400, kindly provided by D. Crane, Griffith University, Brisbane, Australia) (Nguyen et al. 2006; Grant et al. 2013), mouse monoclonal antibodies against α-tubulin (Sigma, T9026) and acetylated α-tubulin (Sigma, T6793) (both used at 1:200 for immunofluorescence, 1:1000 for immunoblotting), and TOM20 (1:200 for immunofluorescence) (BD Transduction Laboratories, San Diego, USA; 612278). Secondary anti-IgG antibodies against rabbit (Alexa 488, 1:500; A21206) and mouse (Alexa 488, 1:400, A21202 and Alexa 594, 1:1000, A21203) were obtained from Molecular Probes (as part of Invitrogen Life Technologies, Eugene, USA). Anti-mouse IgG antibodies conjugated to HRP (1:5000, 170-6516) were obtained from Bio-Rad (Munich, Germany). The specificity of all antibodies has been validated in several previous studies.

### Cell culture and drug treatment

COS-7 cells (African green monkey kidney cells; ATCC CRL-1651) were cultured in DMEM, high glucose (4.5 g/L) supplemented with 10% FBS, penicillin and streptomycin (all from Life Technologies) at 37 °C with 5% CO_2_ and 95% humidity (HERACell 240i CO_2_ incubator). Cells were seeded onto glass coverslips (Fisher Scientific, 19 mm Ø, 0.13–0.17 mm thickness) at a defined density (1 × 10^5^ cells/mL). 24 h after seeding, the culture medium was aspirated, and cells were treated for different time intervals (3, 6 and 24 h) with a range of concentrations of rotenone (Sigma, R8875) (100 nM, 1 µM, 10 µM, 100 µM, 1 mM). For pre-treatment with the microtubule-stabilising drug paclitaxel (taxol) (Sigma, T7402), cells were first incubated with 20 µM paclitaxel for 6 h before the addition of rotenone. For pre-treatment with the antioxidant N-acetyl cysteine (NAC) (Sigma, A7250), cells were first incubated with 10 mM NAC for 1 h before the addition of rotenone. Stock concentrations of 100 mM rotenone and 2 mM paclitaxel were prepared in dimethyl sulfoxide (DMSO) (Sigma, D8418) and dilutions prepared in culture medium. A stock concentration of 100 mM NAC was prepared always fresh in culture medium, and diluted in culture medium following pH neutralisation and sterilisation by filtration of the stock.

### Immunofluorescence and microscopy

Cells grown on glass coverslips were washed twice with phosphate-buffered saline (PBS). Cells to be stained with organelle markers (e.g. PEX14 or TOM20) were fixed using 4% paraformaldehyde, pH 7.4 for 20 min at room temperature as previously described (Bonekamp et al. 2013) as peroxisome morphology is sensitive to alcoholic fixation (Schrader et al. 1995). Cells to be stained with cytoskeletal markers (e.g. α-tubulin or acetylated α-tubulin) were fixed with ice-cold methanol for 15 min at −20 °C. Following fixation, cells were washed 3 times in PBS, membranes were permeabilised with 0.2% Triton X-100 for 10 min at room temperature (permeabilisation was omitted for methanol-fixed cells), washed again three times in PBS and blocked with 2% bovine serum albumin (BSA) for 10 min at room temperature. Cells were then incubated for 1 h with primary antibodies diluted in PBS, washed three times in PBS and incubated for 1 h with secondary antibodies diluted in PBS. In controls, where the primary antibodies were omitted, no staining reactions were observed. To mount coverslips on slides, cells were washed three times in PBS, dipped in dH_2_O and mounted (after removal of excess water) in Mowiol 4-88 containing *n*-propyl galate as an anti-fading (Bonekamp et al. 2013). Cell imaging was performed using an Olympus IX81 microscope with an UPlanSApo 100x/1.40 Oil objective (Olympus Optical, Hamburg, Germany), eGFP ET filter set (470/40 Et Bandpass filter, Beamsplitter T495 LPXR and 525/50 ET Bandpass filter [Chroma Technology GmbH, Olching, Germany]), TxRed HC Filter Set (562/40 BrightLine HC Beamsplitter HC BS 593, 624/40 BrightLine HC [Semrock, Rochester, USA]). Images were taken with a CoolSNAP HQ2 CCD camera (150–300 ms exposure, gain 3, bin 1, gamma 1) and MetaMorph 7 (Molecular Devices, USA) was used to adjust for contrast and brightness.

### Gel electrophoresis and immunoblotting

Following drug treatment, cells were trypsinised, washed in PBS and centrifuged at 500×*g* for 3 min. Cell pellets were lysed [25 mM Tris–HCl, pH 8.0, 150 mM NaCl, 0.5% sodium deoxycholate, 1.5 mM Triton X-100 and a protease-inhibitor mix (Roche Diagnostics)] and protein concentrations were determined using the Bradford assay (Bradford 1976) (Bio-Rad Protein Assay Dye Reagent Concentrate, 5000006). Equal amounts of protein were separated by SDS-PAGE on 12.5% polyacrylamide gels, transferred to nitrocellulose membrane (Amersham Bioscience, Arlington Heights, IL, USA) using a semi-dry apparatus (Trans-Blot SD, Bio-rad) and analysed by immunoblotting using the corresponding primary antibodies and horseradish peroxidase-conjugated secondary antibodies and enhanced chemiluminescence reagents (Amersham Bioscience, Arlington Heights, IL, USA).

### Measurement and quantification of ROS production

Intracellular ROS levels were measured using the fluorescent dye 2′,7′-dichlorodihydrofluorescein diacetate (H_2_DCFDA) (Molecular Probes, Life Technologies). H_2_DCFDA is intracellularly oxidized by ROS, producing the fluorescent compound dichlorofluorescein (DCF), which can be detected by measuring the fluorescence at 530 nm when excited at 485 nm (Fernandez-Gomez et al. 2006; Perez-Ortiz et al. 2004). Cells were seeded in 96-well culture plates (10^4^ cells/well) (Greiner Bio-One) and treated with rotenone after 24 h. At different time points, the medium was removed and the cells were washed one time with PBS. Cells were incubated with 10 µM H_2_DCFDA and the fluorescence intensity was measured immediately every 5 min over a period of 30 min in a multifunctional microplate reader (TECAN i-control—infinite 200, Austria GmbH). An average of 4–6 wells per condition was measured and a mean value obtained. Controls included untreated cells, a blank containing cell culture medium and dye; and H_2_O_2_ together with the dye as a positive control). A linear increase of fluorescence with time was plotted and used to calculate a linear regression. From this, the average relative percentage of ROS production was determined from at least three independent experiments.

### Quantification and statistical analysis of data

Analysis of statistical significance was performed using GraphPad Prism 5 software. A two-tailed unpaired *t* test was used to determine statistical difference against the indicated group. *p < 0.05, **p < 0.01, ***p < 0.001. For analysis of organelle distribution and morphology, a minimum of 150 cells were examined per condition, and organelle parameters (e.g. tubular, elongated morphology, intracellular distribution/clustering) were microscopically assessed in at least three independent experiments. The analysis was made blind and in different areas of the coverslip. Data are presented as mean ± SD.

## Results and discussion

### Rotenone alters peroxisome morphology and distribution

**Fig. 1:**
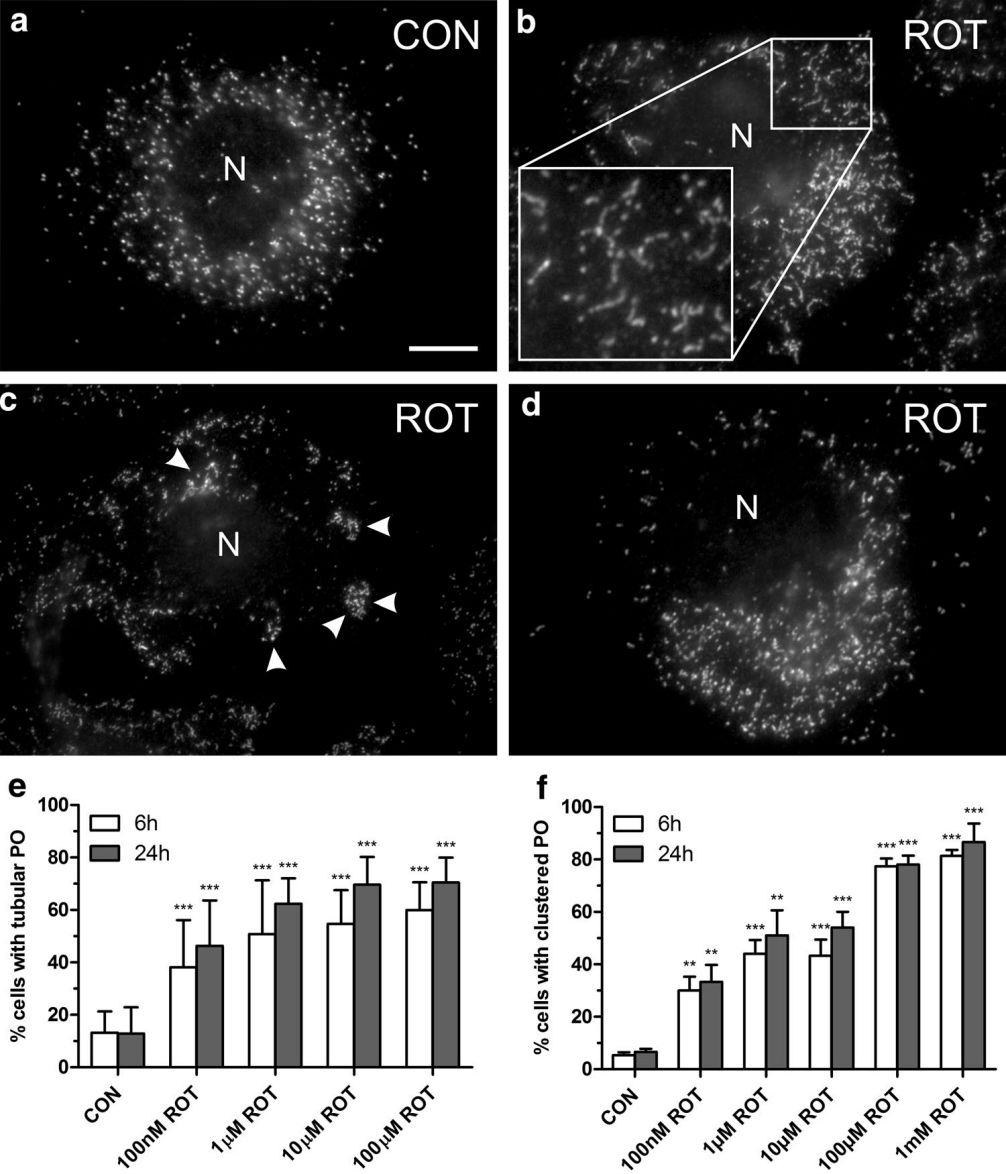
Rotenone induces alterations in peroxisome morphology and distribution. COS-7 cells were treated with solvent (CON) or rotenone (ROT) for 6 and 24 h and processed for immunofluorescence microscopy using antibodies directed to PEX14, a peroxisomal membrane protein. **a–d** Representative examples. **a** Control (CON). Note the elongated tubular peroxisomes in **b** (1 mM ROT, 6 h), the formation of peroxisomal clusters (**c** *arrowheads*) (100 µM ROT, 6 h), and the non-uniform distribution of peroxisomes (**d**) (100 µM ROT, 6 h) in contrast to **a**. Boxed region in **b** shows higher magnification view. **e**, **f** Qualitative assessment of peroxisome elongation (**e**) and cluster formation (**f**). A minimum of 150 cells were examined per condition, and organelle morphology and distribution were microscopically assessed. Values represent mean ± SD of at least three independent experiments. *N* nucleus. *Bar* 10 µm

Rotenone, an agricultural pesticide, is well known to inhibit complex I (NADH CoQ1 reductase) in the mitochondrial respiratory chain (Chance et al. 1963; Higgins and Greenamyre 1996). It is a widely used toxin employed in animal and cellular models of Parkinson’s disease and can freely cross cell membranes (Betarbet et al. 2000; Alam and Schmidt 2002; Mounsey and Teismann 2010). Chronic treatments with rotenone in mice reproduce some features of this disease such as motor deficits, protein aggregation, and loss of dopaminergic neurons (Meurers et al. 2009). Complex I inhibition has several potential functional consequences, including ATP depletion, which in turn induces oxidative stress in cells (Sherer et al. 2003; Testa et al. 2005). To examine whether an inhibitor of the mitochondrial respiratory chain known to produce ROS exhibits an effect on the peroxisomal compartment, COS-7 cells were treated with different concentrations of rotenone (100 nM, 1 µM, 10 µM, 100 µM, 1 mM). Treated cells and controls were processed for immunofluorescence after 6 and 24 h, and peroxisomes were labelled with antibodies against Pex14, a peroxisomal membrane protein (Fig. 1). Peroxisomes in COS-7 cells are uniformly distributed in the cytoplasm, and are usually spherical in shape (Fig. 1a) (Koch et al. 2004; Lin et al. 2016). Treatment with rotenone induced a concentration-dependent elongation of the peroxisomal compartment (Fig. 1b, e) as well as clustering of peroxisomes (Fig. 1c, f) and an uneven distribution of the organelles in the cytoplasm (Fig. 1d). Cells showed either tubular or clustered peroxisomes or a mixture of both. Peroxisome elongation is a pre-requisite of peroxisome multiplication by growth and division (Schrader et al. 2012, 2016), and tubular peroxisomes can be induced by different stimuli including growth factors, fatty acids, and ROS (Schrader et al. 1998, 1999; Schrader and Fahimi 2006). However, peroxisome elongation, clustering and altered distribution have also been observed under conditions of microtubule depolymerisation (Schrader et al. 1996; Wiemer et al. 1997), as peroxisome motility and distribution depend on microtubules in mammalian cells (Schrader et al. 1996, 2003; Wiemer et al. 1997; Lin et al. 2016). Furthermore, rotenone has been reported to inhibit microtubule polymerisation and to arrest cell cycle progression at mitosis (Meisner and Sorensen 1966; Brinkley et al. 1974; Marshall and Himes 1978; Srivastava and Panda 2007). We thus investigated microtubule integrity after treatment of COS-7 cells with rotenone.

### Rotenone exerts a microtubule-destabilising activity in COS-7 cells

**Fig. 2:**
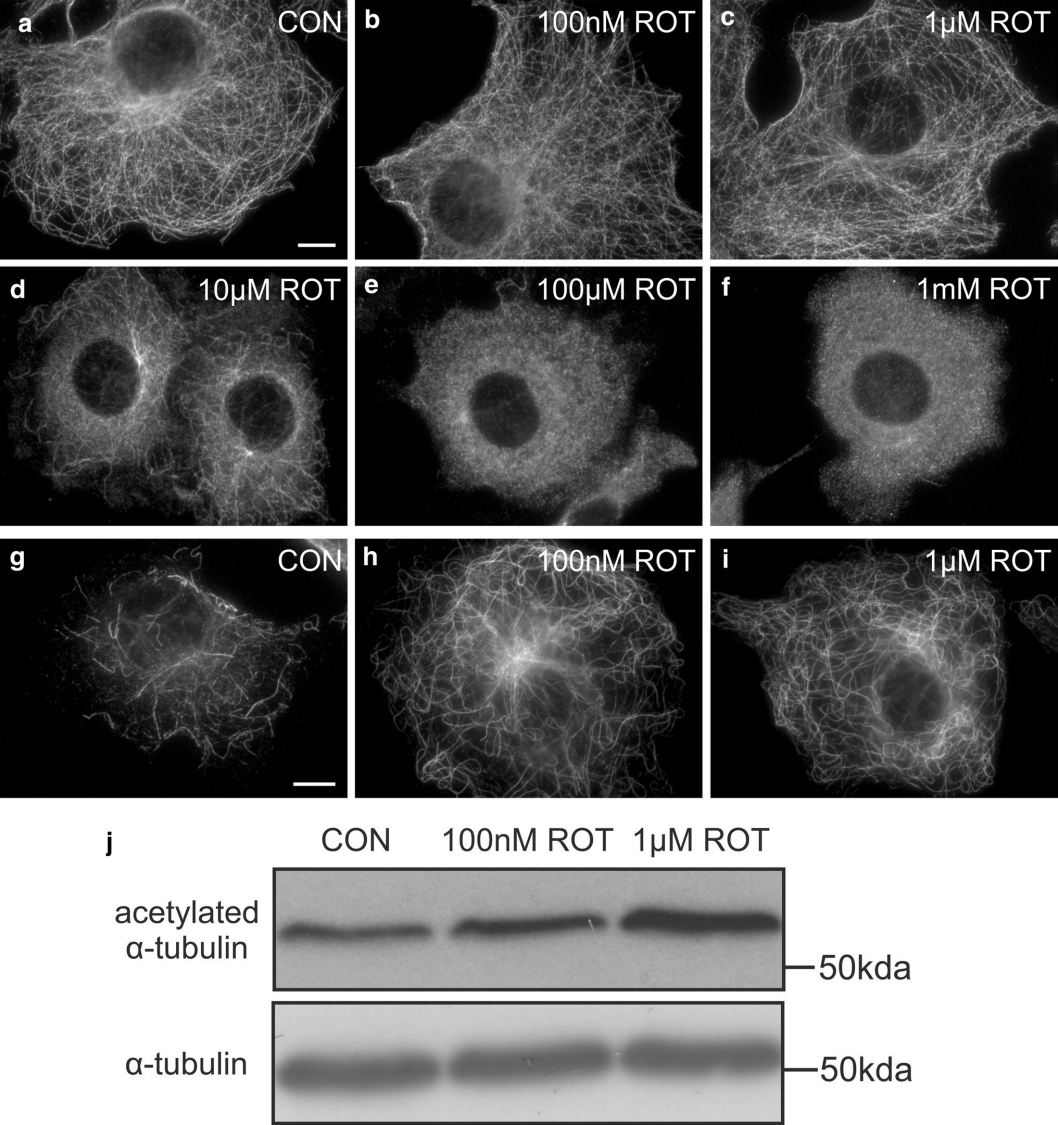
Rotenone induces microtubule depolymerisation and acetylation of α-tubulin in COS-7 cells. **a–f** Cells were treated with solvent (**a**) (CON) or different concentrations of rotenone (ROT) **b–f** for 6 h and processed for immunofluorescence microscopy using antibodies directed to α-tubulin. **g–i** Cells were treated with solvent (**g**) (CON), 100 nM rotenone (**h**) or 1 µM rotenone (**i**) for 3 h and labelled with antibodies directed to acetylated α-tubulin. **j** Immunoblot of cell lysates showing levels of acetylated α-tubulin after 3 h of rotenone treatment. 50 µg of protein was loaded and the blot probed with acetylated α-tubulin and α-tubulin antibody as indicated. α-tubulin serves as loading control. *Bars* 10 µm

COS-7 cells were treated with different concentrations of rotenone and methanol fixed. Controls and treated cells were stained with antibodies to α-tubulin (Fig. 2). In controls, microtubules form the typical radial arrays originating from the microtubule-organising centre (MTOC) in interphase cells (Fig. 2a). At low rotenone concentrations, microtubules were still visible and appeared unaffected (Fig. 2b, c). At an intermediate concentration of 10 µM, some microtubules remained, but the majority was depolymerised (Fig. 2d). Treatment with higher rotenone concentrations resulted in a complete depolymerisation of microtubules (Fig. 2e, f). The effect of rotenone on microtubules was similar after 3, 6 and 24 h.

To investigate microtubule integrity at low rotenone concentrations, microtubules were stained with an antibody to acetylated α-tubulin (Fig. 2g–j). Acetylated α-tubulin is present in various microtubule structures and plays a role in the stabilisation of microtubules (Piperno et al. 1987). In control cells, only a few microtubules were labelled per cell (Fig. 2g). Interestingly, cells treated with low concentrations of rotenone showed a prominent increase in microtubules containing acetylated α-tubulin (Fig. 2h, i). An increase in acetylated tubulin was confirmed by immunoblotting of cell lysates from controls and rotenone-treated cells (Fig. 2j). These findings indicate that although microtubules appear unaffected in cells treated with low concentrations of rotenone based on α-tubulin staining, they already display posttranslational modifications such as acetylation. Tubulin acetylation likely serves to stabilise microtubules and to counteract the destabilising activity of rotenone. It has been suggested that rotenone inhibits microtubule assembly by binding to tubulin, thus inducing a conformational change in tubulin (Srivastava and Panda 2007).

### Alterations in peroxisome distribution induced by rotenone are caused by microtubule depolymerisation

**Fig. 3:**
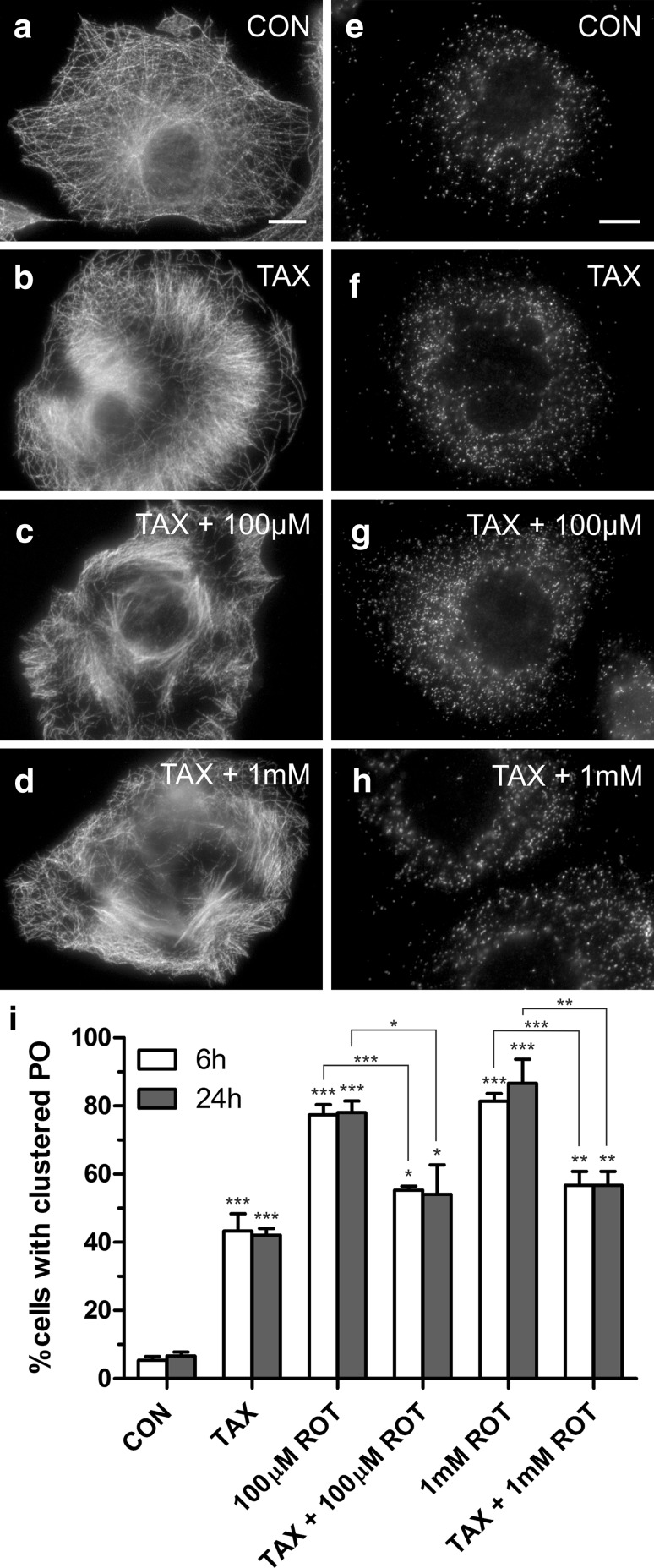
Microtubule stabilisation prevents rotenone-induced clustering of peroxisomes. COS-7 cells were treated with solvent (CON) (**a**, **e**), paclitaxel (TAX) (**b**, **f**), paclitaxel and 100 µM rotenone (ROT) (**c**, **g**), or paclitaxel and 1 mM rotenone (**d**, **h**) for 6 and 24 h. Cells were then processed for immunofluorescence microscopy using antibodies directed to α-tubulin (**a–d**) or PEX14 (**e–h**). **i** Qualitative assessment of peroxisome cluster formation. A minimum of 150 cells were examined per condition, and organelle distribution was microscopically assessed. Values represent mean ± SD of at least three independent experiments. *p* values refer to appropriate controls unless indicated. *Bar* 10 µm

To investigate if peroxisome alterations induced by rotenone are primarily caused by microtubule depolymerisation (or alternatively by Complex I inhibition and oxidative stress), we first pre-incubated COS-7 cells with the microtubule-stabilising agent paclitaxel prior to the addition of rotenone (Fig. 3). Paclitaxel, which causes the formation of microtubule bundles (Fig. 3a, b), protected the microtubules against the microtubule-depolymerising activity of rotenone, even when high concentrations of rotenone were applied (Fig. 3c, d). Interestingly, addition of rotenone after the stabilisation of microtubules with paclitaxel showed no effect on peroxisomes (Fig. 3g–i). Peroxisome distribution was mostly uniform, and clustering was similar to the paclitaxel-treated control (Fig. 3g–i). It should be noted that paclitaxel alone increased cluster formation in COS-7 cells when compared to untreated controls, but this was mainly due to fragmentation of the nucleus, which affected the uniform distribution of peroxisomes in the cytoplasm (Fig. 3b, f). As peroxisome elongation is influenced by many factors (e.g. culture condition, cell density) (Schrader et al. 1996; Schrader and Fahimi 2006), we focussed on the analysis of peroxisomal distribution. However, rotenone-induced peroxisome elongation was no longer observed after pre-treatment with paclitaxel (Fig. 3g, h) and not different from controls (Fig. 3e, f) (unpublished observation). Our findings are in line with previous findings showing that paclitaxel provides protective effects against rotenone-induced toxicity (Jiang et al. 2006). Moreover, paclitaxel had no effect on the morphology and distribution of peroxisomes in HepG2 cells, but pre-treatment with paclitaxel and stabilisation of microtubules prevented alterations of peroxisome morphology and distribution caused by microtubule-depolymerising agents such as nocodazole (Schrader et al. 1996). The results show that the effect of rotenone on peroxisomes in mammalian cells is similar to the action of the microtubule-depolymerising drugs nocodazole, colcemid and vinblastine (Schrader et al. 1996). We suggest that peroxisome alterations in mammalian cells induced by rotenone are mainly caused by the microtubule-destabilising activity of rotenone rather than via complex I inhibition and oxidative stress.

### Rotenone-induced intracellular ROS are not the major cause of peroxisome alterations in COS-7 cells

**Fig. 4:**
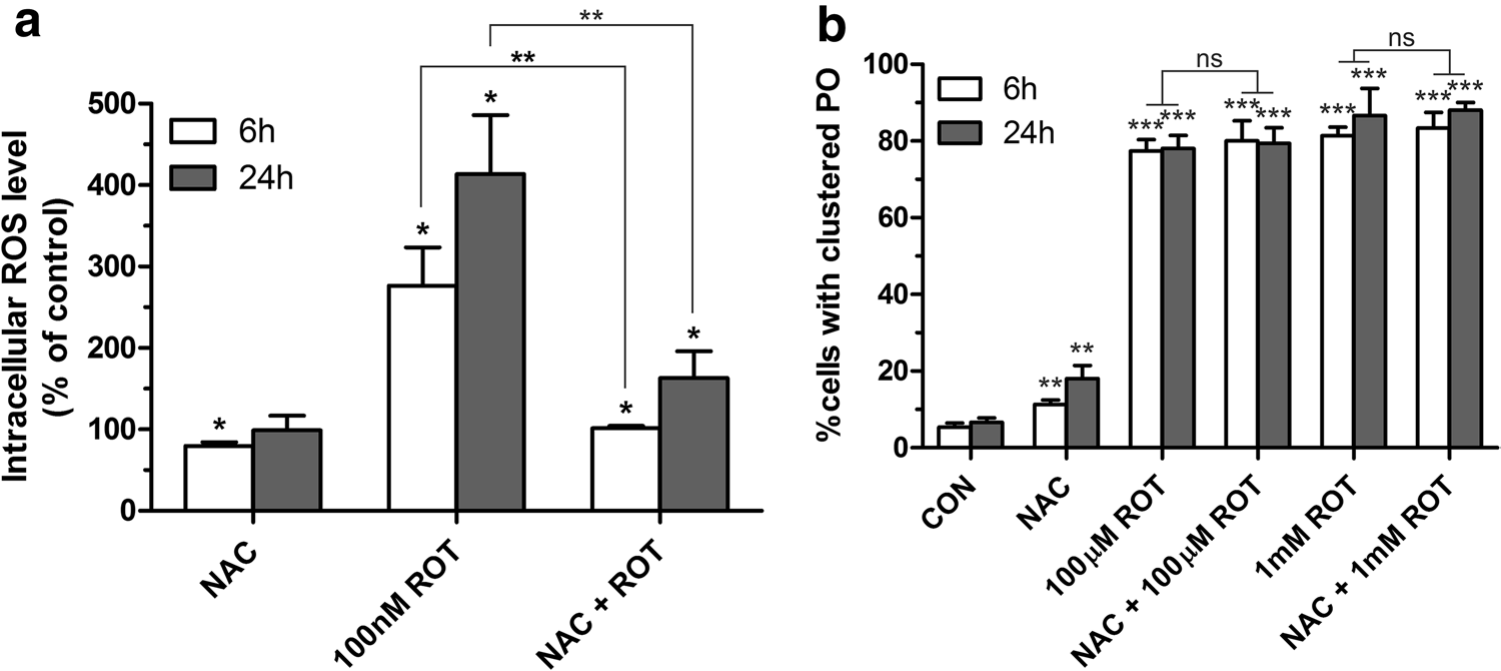
Antioxidant treatment reduces rotenone-induced intracellular ROS levels, but does not prevent peroxisomal clustering. **a** Determination of intracellular ROS levels using the fluorescent dye 2′,7′-dichlorodihydrofluorescein diacetate (H_2_DCFDA) after treatment of COS-7 cells with the antioxidant *N*-acetyl cysteine (NAC), 100 nM rotenone (ROT), or *N*-acetyl cysteine and 100 nM rotenone. Data are expressed as percentage of control (mean ± SD) and are from three independent experiments. **b** Qualitative assessment of peroxisome cluster formation (as described for Fig. 3). Cells were treated as indicated and processed for immunofluorescence microscopy using antibodies directed to PEX14. Values represent mean ± SEM of at least three independent experiments. *p* values refer to appropriate controls unless indicated

Rotenone interferes with the mitochondrial electron transport chain, by inhibiting the transfer of electrons from iron–sulphur centres in complex I to ubiquinone. This prevents NADH from being converted into usable cellular energy (ATP) and, consequently, leads to ROS production. To examine if peroxisome alterations are a consequence of rotenone-dependent ROS production, intracellular ROS levels were measured in COS-7 cells under control conditions and after treatment with 100 nM rotenone (a concentration not affecting microtubule integrity) using 2′7′-dichlorodihydrofluorescein diacetate (H_2_DCFDA) which is intracellularly oxidized by ROS, producing the fluorescent compound dichlorofluorescein (DCF). As shown in Fig. 4a, rotenone increases intracellular ROS levels in COS-7 cells after 6 h (276 ± 6%) and 24 h (414 ± 18%) when compared to control conditions. Furthermore, pre-treatment of COS-7 cells with the antioxidant N-acetyl cysteine (NAC) prior to the addition of rotenone reduced intracellular ROS levels (Fig. 4a). These findings are in line with published results (Bonet-Ponce et al. 2016). More importantly, pre-treatment with NAC prior to the addition of rotenone did not prevent clustering of peroxisomes and changes in the uniform distribution of the organelle (Fig. 4b). NAC alone only induced a slight increase in peroxisomal clusters compared to control conditions. These observations support our assumption that peroxisome alterations in mammalian cells induced by rotenone are mainly caused by the microtubule-destabilising activity of rotenone and not via complex I inhibition and increased intracellular ROS levels.

### Rotenone alters mitochondrial morphology in a ROS dependent manner

**Fig. 5:**
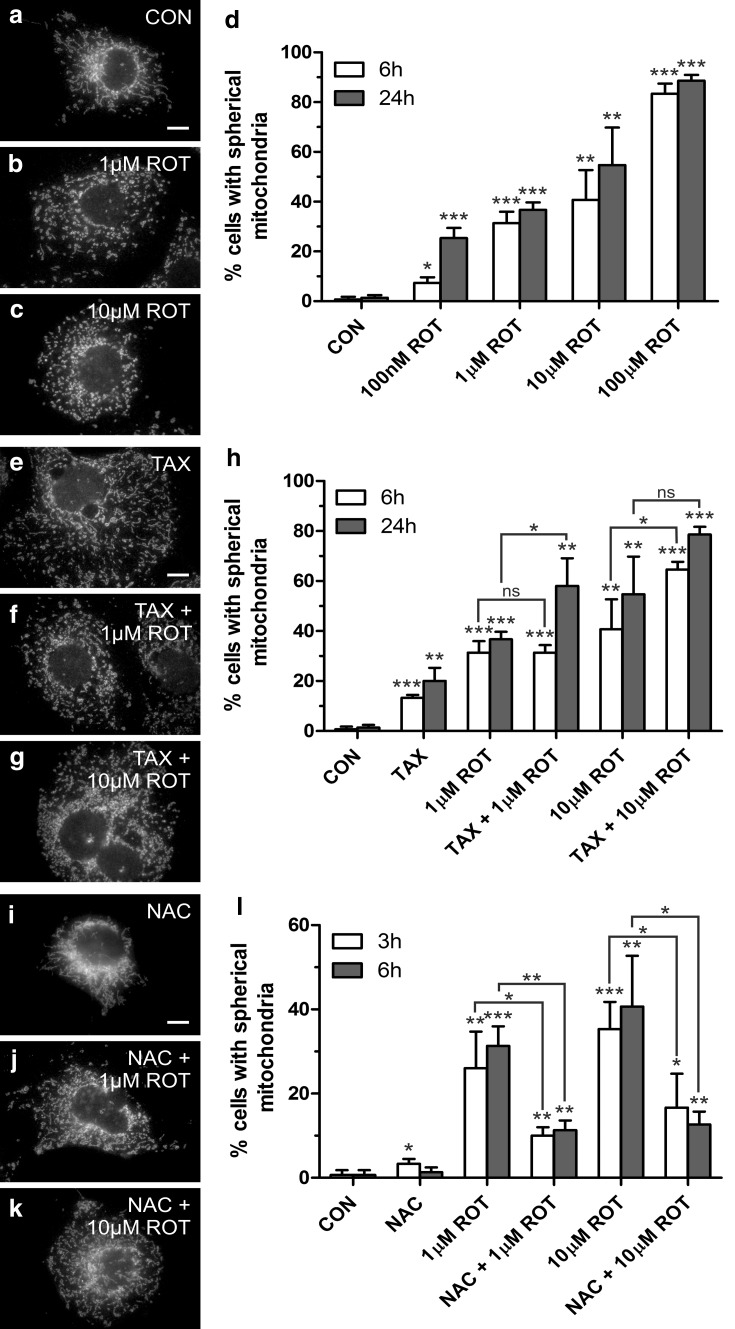
Rotenone-induced mitochondrial fragmentation can be prevented by antioxidant-treatment but not by microtubule stabilisation. **a–d** Rotenone induces the formation of spherical mitochondria. COS-7 cells were treated with solvent (CON) (**a**) or rotenone (ROT) for 6 (**b**–**d**) and 24 h (**d**) and processed for immunofluorescence microscopy using antibodies directed to TOM20, a mitochondrial outer membrane protein. Note the elongated tubular mitochondria in **a** in contrast to **b**, **c**. **d** Qualitative assessment of mitochondrial morphology. A minimum of 150 cells were examined per condition, and organelle morphology was microscopically assessed. Values represent mean ± SD of at least three independent experiments. **e–h** Microtubule stabilisation does not prevent rotenone-induced mitochondrial fragmentation. COS-7 cells were treated with paclitaxel (TAX) (**e**) or paclitaxel and rotenone (ROT) for 6 (**f**–**h**) and 24 h (**h**) and labelled with TOM20 antibodies. Note the elongated tubular mitochondria in **e** in contrast to **f**, **g**. **h** Qualitative assessment of mitochondrial morphology (see above). **i–l** Antioxidant-treatment prevents rotenone-induced mitochondrial fragmentation. COS-7 cells were treated with *N*-acetyl cysteine (NAC) (**i**) or *N*-acetyl cysteine and rotenone (ROT) for 3 (**j–l**) and 6 h (**l**) and labelled with TOM20 antibodies. Note the elongated tubular mitochondria in **j–l** in contrast to **b**, **c**, **f**, **g**. **l** Qualitative assessment of mitochondrial morphology (see above). *p* values refer to appropriate controls unless indicated. *Bars* 10 µm

Next, we investigated the morphology of mitochondria in COS-7 cells treated with different concentrations of rotenone. Treated cells and controls were processed for immunofluorescence, and mitochondria were labelled with antibodies against TOM20, a mitochondrial outer membrane protein (Fig. 5). Mitochondria in COS-7 cells usually display an elongated, tubular and bulbous morphology (Fig. 5a, e, i) (Koch et al. 2004). In contrast to its elongating effect on peroxisomes (Fig. 1), rotenone induced a concentration-and time-dependent fragmentation of mitochondria resulting in a spherical, round organelle phenotype (Fig. 5b–d). Pre-treatment of COS-7 cells with paclitaxel to stabilise microtubules did not prevent mitochondrial fragmentation (Fig. 5e–h). However, pre-treatment of COS-7 cells with the antioxidant NAC prevented mitochondrial fragmentation even at high rotenone concentrations and maintained a tubular mitochondrial morphology (Fig. 5i–l). These findings indicate that mitochondrial alterations in mammalian cells induced by rotenone are mainly caused by complex I inhibition and increased intracellular ROS levels rather than via the microtubule-destabilising activity of rotenone, which is in contrast to its action on peroxisomes.

We conclude from these observations that at low concentrations (100 nM–10 µM) rotenone acts on mitochondria in COS-7 cells and induces their fragmentation, likely due to the inhibition of complex I and subsequent oxidative stress/increased intracellular ROS levels. Microtubules at these concentrations are still intact (Fig. 2), although exhibiting increased levels of acetylated α-tubulin (Fig. 2). Alterations in peroxisome morphology and distribution are more prominent at higher rotenone concentrations, as they are induced by the microtubule-depolymerising activity of rotenone, and not primarily via increased intracellular ROS levels.

Our findings support published data indicating that mitochondria are more susceptible to changes in cellular redox homeostasis and increased ROS levels than peroxisomes. For example, the neurotoxin 6-hydroxydopamine, which is supposed to cause ROS generation (Galindo et al. 2003), induced a profound mitochondrial fragmentation in SH-SY5Y neuroblastoma cells, but failed to induce any changes in peroxisome morphology (Gomez-Lazaro et al. 2008). In line with this, we observed no effect on peroxisome morphology when other inhibitors of mitochondrial respiration were used (e.g. sodium azide, an inhibitor of cytochrome c oxidase/complex IV) (unpublished observations). Furthermore, peroxisomes were found to resist oxidative stress generated elsewhere in the cell, whereas generation of excess ROS inside peroxisomes perturbed the mitochondrial redox balance and led to mitochondrial fragmentation and cell death (Ivashchenko et al. 2011; Wang et al. 2013). How redox communication between peroxisomes and mitochondria is mediated is currently unclear, but may involve a more complex signalling system as opposed to simple diffusion of excess ROS (Lismont et al. 2015).

Our data also indicate that in cellulo and in vivo studies with rotenone, which has been widely used to study Parkinson’s disease (Betarbet et al. 2000), have to be carefully interpreted due to the different effects of rotenone in mammalian cells. We show that besides increasing intracellular ROS levels, mitochondrial fragmentation, and microtubule depolymerisation, rotenone also has an effect on peroxisome dynamics and distribution. Such processes are important for the formation of peroxisomes by growth and division, their intracellular positioning, movement and interaction with other subcellular compartments and have been linked to human health and disease (Schrader et al. 2015a; Ribeiro et al. 2012). Mitochondrial dysfunction is commonly accepted as having a key role in Parkinson’s disease; however, microtubule alterations are also considered. For example, 1-methyl-4-phenyl-1,2,3,6-tetrahydropyridine (MPTP), another model toxin for Parkinson’s disease, impacts on microtubule polymerisation besides its effect on mitochondria (Cartelli et al. 2010). Disrupting the microtubule network can alter vesicular and organelle transport in neurons leading to neuronal death (Ren et al. 2005; De Vos et al. 2008; Cartelli et al. 2010). Loss of peroxisome dynamics and distribution may contribute to this process.

## Abbreviations

NAC: *N*-Acetyl cysteine
ROS: Reactive oxygen species
ROT: Rotenone
TAX: Paclitaxel
